# Cardiac Signatures of Personality

**DOI:** 10.1371/journal.pone.0031441

**Published:** 2012-02-21

**Authors:** Stefan Koelsch, Juliane Enge, Sebastian Jentschke

**Affiliations:** Cluster of Excellence “Languages of Emotion”, Freie Universität Berlin, Berlin, Germany; Université de Montréal, Canada

## Abstract

**Background:**

There are well-established relations between personality and the heart, as evidenced by associations between negative emotions on the one hand, and coronary heart disease or chronic heart failure on the other. However, there are substantial gaps in our knowledge about relations between the heart and personality in healthy individuals. Here, we investigated whether amplitude patterns of the electrocardiogram (ECG) correlate with neurotisicm, extraversion, agreeableness, warmth, positive emotion, and tender-mindedness as measured with the Neuroticism-Extraversion-Openness (NEO) personality inventory. Specifically, we investigated (a) whether a cardiac amplitude measure that was previously reported to be related to flattened affectivity (referred to as 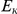 values) would explain variance of NEO scores, and (b) whether correlations can be found between NEO scores and amplitudes of the ECG.

**Methodology/Principal Findings:**

NEO scores and rest ECGs were obtained from 425 healthy individuals. Neuroticism and positive emotion significantly differed between individuals with high and low 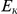 values. In addition, stepwise cross-validated regressions indicated correlations between ECG amplitudes and (a) agreeableness, as well as (b) positive emotion.

**Conclusions/Significance:**

These results are the first to demonstrate that ECG amplitude patterns provide information about the personality of an individual as measured with NEO personality scales and facets. These findings open new perspectives for a more efficient personality assessment using cardiac measures, as well as for more efficient risk-stratification and pre-clinical diagnosis of individuals at risk for cardiac, affective and psychosomatic disorders.

## Introduction

The personality of an individual has profound effects on the peripheral physiology. Such effects appear to be due to the modulatory influence of brain structures implicated in personality (such as the orbitofrontal cortex, amygdala, insular cortex, and hippocampal formation) [1], [2], [3], [4], [5], [6], [7] on peripheral organs and tissues through the autonomic, the endocrine, and the immune system [2], [8], [9], [5], [10], [11], [7], [12], [13]. These modulatory influences are relevant for the understanding of a wide range of somatic diseases, such as cardiovascular disease [14], [15], inflammatory disorders [16], or autoimmune diseases [17].

**Figure 1:**
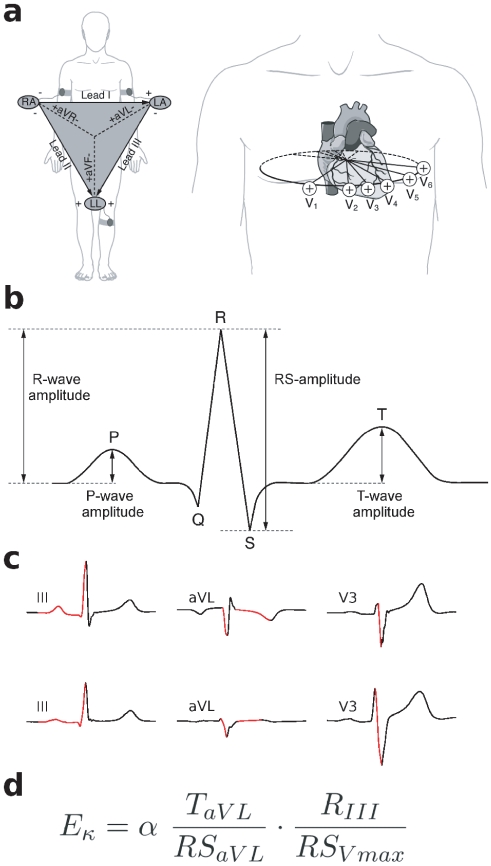
Illustration of ECG waves and 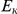 values. (**a**) Illustration of standard ECG leads: The six extremity leads (I, II, III, aVL, aVR, aVF) record voltage differences by means of electrodes placed on the limbs (left panel). The triangle shows the spatial relationships of the extremity leads, which record electrical voltages onto the frontal plane of the body. The six chest leads (V1–V6) record voltage differences by means of electrodes placed on the chest wall (right panel). The oval indicates spatial relationships of the six chest leads, which record electrical voltages transmitted onto the horizontal plane. (**b**) Illustration of ECG amplitude parameters used in the present study (P-, R-, RS-, and T-waves, each wave is measured from each of the twelve ECG leads in each individual). Due to the absence of a Q-wave in some individuals at some leads, the R-wave is measured with regard to the iso-electric line. (**c**) Illustration of ECG amplitude parameters used to calculate 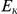 values (indicated in red): R-wave of III (measured from the baseline preceding the P-wave), RS-complex of aVL, T-wave of aVL, and RS-complex of the chest lead with the maximal RS amplitude (usually V3 or V2). The upper panel shows averaged ECG cycles measured from a male subject with a high 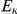 value (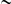
90th percentile of our study population), the lower panel shows averaged ECG cycles from a male subject with a low 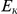 value (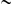
10th percentile of our study population). Both subjects had similar body height (184 vs. 185 cm), body weigt (70 vs. 72 kg), and normal QRS axis orientation (96.23 vs. 75.24). Note the smaller T-wave in aVL in relation to the RS-wave in aVL, and the smaller R-wave in III in relation to the RS-wave in V3, in the subject with low 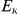 value (lower panel), compared to the subject with high 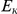 value (upper panel). (**d**) Equation for computation of 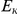 values. Computation of the ECG amplitude parameters shown in (**c**) leads to an 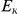 value of 1.61 for the ECG shown in the upper, and an 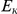 value of 0.17 for the ECG shown in the lower panel of (**c**) (for a better readability, values are scaled by a factor 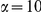). Abbrevations: 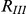: R-wave of lead III (measured from the baseline preceding the P-wave); 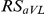: RS-complex of aVL; 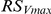: RS-complex of the chest lead with the maximal RS amplitude; 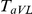: T- wave of aVL.

The present study uses electrocardiography to investigate such modulatory influences of personality traits on peripheral physiology in healthy individuals. Usually, electrocardiograms (ECGs) are recorded with several leads and a temporal resolution in the millisecond range, thus providing multidimensional information about cardiac function (see also Figure 1a,b). Regional cardiac function (such as conduction of excitation, conduction velocity, contractile force, coronary circulation, as well as aspects of cardiac valve function) is modulated by neurons in intrathoracic extracardiac and intrinsic cardiac ganglia [18]. These ganglia probably represent the final common pathway through which the diverse, extrinsic neural signals to the heart are modified before being transmitted to the effector tissues [19]. The neurons of these ganglia form the cardiac nerve plexus, a system of nerve cells within and around the heart which integrates and modifies sensory input and cardiac output [19].

Other than traditionally believed, intrathoracic ganglia are not merely relay stations for autonomic efferent neuronal control of the heart, but integrate the spatial and temporal summation of varied sensory inputs, as well as inputs from central sympathetic/parasympathetic nerve fibres [19]. Therefore, activity of neurons within the cardiac nerve plexus (and thus regional heart activity) is directly and indirectly modulated by several psychological factors: (1) Both parasympathetic and sympathetic nerve fibres project into the cardiac nerve plexus [20]. Such autonomic activity is an integral component of emotion and affective traits [14], [21]. (2) Efferent autonomic neuronal activity is modulated by limbic/paralimbic forebrain structures such as hypothalamus, amygdala, insular cortex, and (medial) orbitofrontal cortex. The orbitofrontal cortex, e.g., hosts neurons projecting directly to the the parabrachial nucleus, the nucleus of the solitary tract, and to sympathetic preganglionic neurons in the interomedial lateral nucleus of the spinal cord [20]. This is relevant because patients with orbitofrontal lesions show marked personality changes [3], lending plausibility to the assumption that personality has peripheral physiological effects that become apparent in regional activity of the heart. (3) The left and right hemispheres of the human forebrain are assumed to have different associations with particular emotions and affective traits [21], probably due to an asymmetrical representation of homeostatic activity that originates from asymmetries in the peripheral autonomic nervous system. Lesion studies indicate a right hemisphere dominance for sympathetic efferent neuronal effects, and stimulation of the right anterior insular cortex elicits increases in heart rate and blood pressure, while left anterior insular stimulation decreases heart rate [22], [23], [20]. Correspondingly, different emotional states and affective traits exert asymmetric autonomic outflow that can modulate regional activity of the heart. For example, the right cardiac nerve innervates the anterior surface of the heart (including the sinoatrial node, the atrioventricular node, and the anterior surfaces of the right and left ventricles), whereas the left cardiac nerve innervates the posterolateral surface of the heart (including the atrioventricular node and the posterior surfaces of the right and left ventricles) [24], [25]. (4) Activity of neurons within intrathoracic autonomic ganglia is modulated by circulating hormones (in particular circulating adrenalin and angiotensin II), as well as by other sensory information (such as vascular information, information about blood pressure, electrolytic balance, and blood gases). Due to the effects of particular emotions and affective traits on circulating hormones (including adrenalin), blood pressure and blood gases, such emotions and affective traits indirectly also modulate regional activity of the heart.

In clinical populations, the diverse effects of affective traits on the heart have been demonstrated by a plethora of clinical and experimental evidence implicating anger, hostility, depression and anxiety in the occurrence of arteriosclerosis, coronary artery disease, hypertension, myocardial ischemia and infarction, cardiac arrythmia formation, and sudden cardiac death [26]. Our approach is to find correlations between regional heart activity (as reflected in the various waves of different ECG leads) and affective traits in non-clinical populations.

The present study builds upon a previous study that used high-resolution ECGs to investigate whether amplitude patterns of ECG waves correlate with flattened affectivity [6]. In that study [6], flattened affectivity was assessed based on interviews, and characterized by emotional coldness and a limited capacity to express warm, tender feelings (as indicated by reduced facial and eye expression of emotion, reduced prosodic and gestural expression of emotion, and sparse use of emotional language) [27], [28]. Using discriminant analysis, four ECG waves were found as predictors for flattened activity (Figure 1c). The computation of these values according to the equation shown in Figure 1d results in a single value (henceforth referred to as 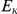 value) for each individual. 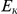 values were lower in individuals with flattened affectivity than in normal controls, and differences in 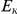 values were related to autonomic (sympatho-vagal) balance: Individuals with low 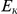 values showed a lower heart rate variability (HRV, in terms of a lower variability of beat-to-beat intervals), as well as lower high-frequency (HF) and higher low-frequency (LF) spectral power (and a higher LF/HF ratio) than subjects with high 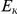 values [6]. Moreover, two functional neuroimaging experiments showed functional differences in the amygdala and the hippocampal formation between individuals with high and low 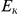 values [6], supporting the notion of differences in emotionality between groups.

The first aim of the present study was to validate the relation between 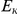 values and emotional personality using standardized personality measures. Therefore, we investigated whether scores of neuroticism, extraversion, and agreeableness (as measured with the Neuroticism-Extraversion-Openness Five Factor Inventory, NEO-FFI) [29], as well as scores of positive emotion, warmth, and tender-mindedness (as measured with the Revised NEO Personality Inventory, NEO-PI-R) [29] differed between individuals with higher and lower 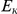 values. The facets positive emotion (i.e., the tendency to experience positive emotions), warmth (i.e., interest in and friendliness towards others), and tender-mindedness (i.e., attitude of sympathy for others) [29] were chosen because they are related to the concept of flattened affectivity [6]. These three facets are part of the personality factors extraversion and agreeableness, of which we obtained the entire scales, along with the neuroticism scale (which is also related to the experience of emotional states), using the NEO-FFI [29]. In specific, we hypothesized that individuals with low 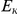 values would show higher neuroticism scores, and lower scores of extraversion, agreeableness, warmth, tender-mindedness, and positive emotion. This would represent further evidence for a relation between ECG amplitude ratios (as measured with the 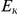 values) and personality traits. In addition, based on previous results [6], individuals with low 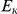 values were expected to show a lower HRV (in terms of beat-to-beat interval variability), as well as lower HF and higher LF spectral power (and a higher LF/HF ratio) than subjects with high 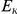 values.

Furthermore, the present study also takes a new approach: We investigated whether the scores of the NEO scales (neuroticism, extraversion, and agreeableness) and NEO facets (positive emotion, warmth, and tender-mindedness) can be used to identify cardiac amplitude signatures related to an individual's personality. Therefore, we obtained the amplitude values of the ECG waves shown in Figure 1b from each subject, separately for each of the twelve ECG leads (leading to an array of 48 values per subject). Using stepwise regressions, we then investigated whether an individual's personality (as measured with the NEO) can be predicted based on the ECG amplitude pattern of that individual.

The rationale for our approach is that personality traits have peripheral-physiological effects which are reflected in the activity of the heart. Previous work with non-clinical adult populations suggests relations between temporal measures of heart activity, showing lower HRV in individuals with higher neuroticism [30], consistent with findings of lower HRV in depressive patients (without myocardial infarction) [31]. However, reports on relations between personality and HRV in non-clinical populations are extremely sparse, and the relatively moderate test-retest reliability of HRV measures renders HRV measures suboptimal for the assessment of personality aspects (this is partly due to HRV being influenced, for example, by the breathing rate and the circadian rhythm) [32]. The test-retest reliability of 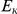 values, by contrast, appears to be relatively high (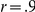 in our previous study) [6]. Moreover, compared to personality questionnaires, physiological measures do not face the problem of potential subjective bias such as socially desirable responding, inaccuracies in self-perception, self-favouring tendencies, self-deception, and moralistic bias [33], [34], [35], [36], [37], and may thus be better suited to assess long-term emotional dispositions related to personality. Therefore, the discovery of ECG signatures reflecting personality traits would not only broaden our knowledge about cardiac effects of personality, but also represent a step towards the diagnostics of emotional personality aspects with cardiac measures.

## Methods

### Subjects

425 participants (212 females), aged between 18 and 33 years, were measured. All participants were university students. Exlusion criteria were previous diagnosis of any cardio-vascular disease, or any mental or psychiatric disorder (no clinical diagnostic interviews were conducted in the course of this study). Written informed consent was obtained, the study was approved by the ethics committee of the University of Leipzig, and conducted according to the guidelines of the Declaration of Helsinki.

### Questionnaires

We obtained scores of the personality scales *Extraversion*, *Agreeableness* and *Neuroticism* (each with 12 Items) from the German translation of the NEO Five Factor Inventory (NEO-FFI) [29], [38], as well as scores of the personality facets *warmth*, *positive emotion* and *tender-mindedness* (each with 8 Items) from the German translation of the revised version of the NEO Personality Inventory (NEO-PI-R) [29], [39]. These facets were used because they are more closely related to the construct of flattened affectivity than other NEO-PI-R facets (*positive emotion* is related to a tendency to experience positive emotions, *warmth* is related to interest in and friendliness towards others, and *tender-mindedness* is related to an attitude of sympathy for others) [29]; thus, these facets appeared to be well suited to test whether 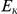 values are related to personality facets. The number of items amounted to 54 Items; note that the NEO-FFI is a short version of the NEO-PI-R and that, hence, twelve items of the NEO-FFI scales are identical to those of the NEO-PI-R facets (warmth and positive emotion are facets of the extraversion scale, and tendermindedness is a facet of the agreeableness scale). Each item was answered on a five-steps Likert scale (“strongly disagree”, “disagree”, “neutral”, “agree”, “strongly agree”). These response categories were re-coded into values from 0 to 4 (with 4 corresponding to a high score of the respective scale of the facet); then for each subject the summed values for each facet or scale were divided by the number of items (thus a score of 0 indicates the lowest possible score for a facet or scale, and a score of 4 indicates the highest possible score for a scale or facet). For each individual, all scores were then standardized according to the manuals of the German version of the NEO-FFI [38] and the NEO-PI-R [39].

### ECG Measurements and Data analysis

From each participant, a 12 leads resting ECG (2 min duration) was obtained in supine position under standard conditions according to the guidelines of the Task Force of the European Society of Cardiology and the North American Society of Pacing and Electrophysiology [32]. ECGs were recorded (with disposable electrodes) with high resolution (sampling rate: 1000 Hz, resolution: 22 bit, without on-line filtering) using a Refa-system (Twente Medical Systems, Enschede, NL).

All ECG analyses were carried out blinded, that is, without any knowledge about the NEO-scores of any participant. ECG-wave detection and measurement of ECG amplitude values was performed electronically (using the in-house software package Kardionoon 1.0) [6] and visually controlled by the first author. The amplitude measurements are described in detail elsewhere [6]; in short, for each lead of each participant, all P-, R-, S-, and T-wave peaks were identified in the raw ECG, and artifact-free waves were then averaged separately for each lead to obtain ECG waves representative for each participant (i.e., for an artifact-free ECG in a subject with a heart rate of 60 beats per minute, 120 P-, 120 R-, 120 R-S-, and 120 T-waves were averaged, separately for each wave, and for each of the twelve ECG leads). From these averaged ECG waves, absolute amplitude values of P-, R-, R-S-, and T-waves were measured electronically; R-wave amplitudes were measured with respect to the baseline of the averaged ECG cycle (see also Figure 1b for illustration), T-wave amplitudes were measured with respect to the first plateau-like wave shape preceding the T-wave peak [6]. Moreover, to reduce potential bias introduced by slightly different placement of chest electrodes, the maximal value of each of the four ECG waves (P-, R-, R-S-, and T-wave) measured at any of the chest leads was entered into further analysis. Thus, an array of 52 ECG amplitude values was obtained from each individual (4 ECG amplitude values×12 ECG leads, plus the 4 maximal ECG wave values measured at the chest leads).

With regard to the HRV, the following temporal and spectral measures of the HRV were electronically computed and analyzed for each subject (using the BIOSIG toolbox) [40]: mNN (mean time of successive heart beat intervals), SDNN (SD of successive heart beat intervals), RMSSD (root mean square of the SD of successive heart beat intervals), SD1, SD2, low frequency power (LF, 0.04–0.15 Hz), high frequency power (HF 0.15–0.4 Hz), and the LF/HF ratio (both LF and HF were calculated in normalized units, n.u.). QRS detection was visually controlled by the first author.

The 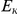 value was computed for each subject according to the equation shown in Figure 1d. To test whether NEO personality scores, as well as HRV values, differ between individuals with higher and lower 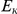 values, subjects were divided into two groups based on a median split of the 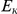 values. Subsequently, scores of NEO-scales and NEO-facets, as well as HRV measures (mNN, SDNN, RMSSD, SD1, SD2, LF n.u., HF n.u., LF/HF) were compared between groups using two-sided independent samples 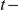
tests. Test were carried out according to the directed hypotheses based on our previous study [6], therefore the significance level was set to 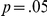.

To investigate correlations between ECG amplitude values and body height, body weight, and body mass index (BMI), stepwise linear regressions were performed with height, weight, and BMI as dependent variables, and all ECG amplitude values (52 per subject, see above) as independent variables. 32 participants were excluded from this analysis, because not all amplitude values could be obtained (e.g., no clear P- or T-wave could be measured at aVL), resulting in a pool of 393 participants (194 males) used for the regression analyses. To investigate correlations between personality scores of the NEO scales and ECG amplitude values, stepwise linear regressions were performed for each scale and facet separately, with the standardized scores of each score, and the T-scores of each facet, as dependent variable, and all ECG amplitude values as independent variables; in addition, body height, body size, and BMI were included as independent variables (to estimate a possible influence of these variables). Stepwise regressions were calculated using using SPSS 19 (IBM, NY). To ensure the validity of the results obtained from the stepwise regression analyses, cross-validated (leave-one-out) regression coefficients were computed using matlab (Nattick, MA): For each participant a predicted value was calculated using the b-weights from the stepwise regression of all remaining participants. That is, 393 stepwise regressions were performed, each without the data set of one individual (‘leave-one-out’), in order to use the regression model obtained with the remaining 392 individuals to predict the score of the ‘left-out’ individual). Then, the correlation between the predicted scores and the actual scores was calculated. This cross-validated regression was performed to ensure that any statistically significant regression would not be due to the large number of subjects, or the large number of variables used for the regressions. Bonferroni-corrected significance level was 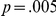.

## Results

### Analysis of 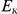 values

**Figure 2:**
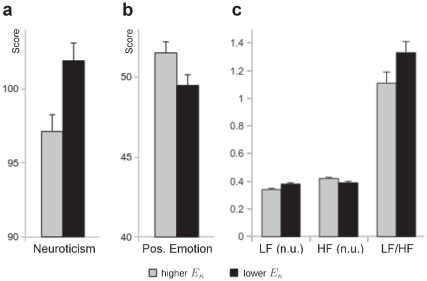
Group comparisons between individuals with 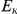 values above and below the median of 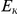 values. Individuals with higher 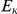 values had lower neuroticism (**a**), and higher positive emotion scores (**b**). Moreover, individuals with higher 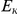 values had lower normalized LF power, higher normalized HF power, and a lower LF/HF ratio (**c**). Abbrevations: LF (n.u.): low frequency power (0.04–0.15 Hz) of the HRV in normalized units; HF (n.u.): high frequency power (0.15–0.4 Hz) of the HRV in normalized units; LF/HF: ratio of low frequency power to high frequency power.

Based on the median of the 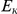 values (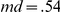, range: 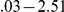), participants were split into two groups (higher and lower 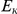 values) to compare NEO scores and HRV parameters between groups. Two-sided independent samples 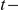
tests indicated that individuals with lower 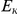 values showed significantly higher neuroticism (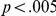, Cohen's 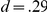, Figure 2a) and lower positive emotion (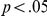, Cohen's 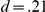, Figure 2b), and there was a trend for lower extraversion in individuals with lower 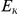 values (see Table 1a for complete statistics). Moreover, with regard to the HRV values, analogous 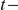
tests indicated that individuals of the group with lower 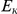 values had lower HF (normalized units) power (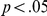, Cohen's 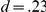), higher LF (normalized units) power (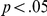, Cohen's 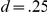), and a higher LF/HF ratio (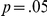, Cohen's 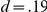, see also Table 1b, Figure 2c), and there was a trend for a lower mean heart rate in the group with lower 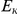 values.

**Table 1:** NEO-scores, heart rate variability values, and body characteristics (means, with SD in parentheses), separately for the group with 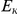 values below and above the median of 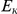 values.

	lower 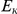	higher 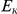	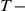 ( 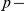 ) values	Cohen's 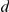
**(a)** NEO-parameters				
Neuroticism	101.88 (1.20)	97.11 (1.13)	**−2.88 (.004)**	−.29
Extraversion	100.85 (1.31)	104.36 (1.23)	1.95 (.052)	.19
Agreeableness	103.81 (1.14)	106.00 (1.11)	1.38 (.168)	.14
Warmth	50.30 (.71)	51.90 (0.75)	1.54 (.123)	.15
Positive Emotion	49.48 (.66)	51.51 (0.69)	**2.13 (.034)**	.21
Tender- mindedness	48.49 (.72)	49.22 (0.69)	0.723 (.470)	.07
**(b)** HRV-parameters				
mNN	921.35 (9.46)	950.98 (11.97)	1.95 (.052)	.19
RMSSD	70.23 (4.07)	71.26 (4.35)	.173 (.863)	.02
LF (n.u.)	.38 (.01)	.34 (.01)	**−2.53 (.012)**	−.25
HF (n.u.)	.39 (.01)	.42 (.01)	**2.33 (.020)**	.23
LF/HF	1.33 (.08)	1.11 (.08)	**−1.97 (.050)**	−.19
**(c)** Body characteristics				
Age (yr)	24.62 (.21)	24.47 (.17)	0.55 (.581)	−.05
Height (m)	1.76 (.65)	1.75 (.68)	1.02 (.310)	−.09
Weight (kg)	69.73 (.85)	68.58 (.81)	.98 (.326)	−.09
BMI	22.52 (.20)	22.40 (.18)	.43 (.670)	−.04

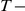
 and 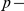
values indicate results of independent samples 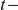 tests, significant test results are indicated by bold font. The outermost right column provides effect sizes as indicated by Cohen's 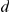 (Hedges' bias correction, values >.2 indicate moderate effect sizes). Abbrevations: BMI: body mass index (body weight/body height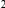); HF (n.u.): high frequency power (0.15–0.4 Hz) of the HRV in normalized units; HRV: heart rate variability; LF (n.u.): low frequency power (0.04–0.15 Hz) of the HRV in normalized units; RMSSD: root mean square of the SD of successive heart beat intervals.

Body height, body weight, and BMI was virtually identical in both groups (see Table 1c for statistics). Likewise, QRS axis orientation was similar in both groups: Median of QRS axis orientation was 67.01 in the group with higher, and 64.43 in the group with lower 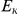 values. A Mann-Whitney 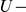
test did not indicate a significant difference of QRS axis orientation between the group with higher and lower 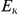 values (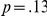; a non-parametric test was used because values of QRS axis orientation were not normally distributed according to a Kolmogorov-Smirnov test, 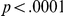).

We also compared the scores of each NEO item between groups to further characterize personality differences between individuals with low and high 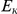 values. Six items were identified (see Table S4) for which the scores differed between groups with effect sizes of Cohen's 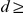.2 (Hedges' bias correction; when comparing the scores of these items between groups using two-samples 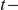
tests, 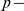
values were 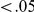 in each of the tests). These items were characterized by words such as ‘light-hearted’, ‘cheerful optimism’, ‘tense and jittery’, happiness', ‘lonely or blue’, and ‘high-spirited’.

### Regressions between NEO personality parameters and ECG amplitudes

**Figure 3:**
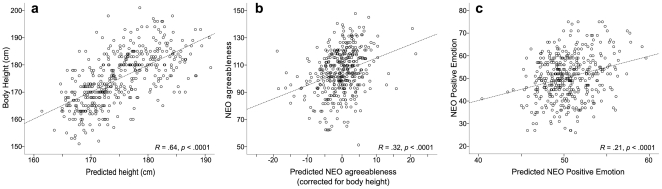
Stepswise regressions. (**a**) Result of the stepwise regression with body height as dependent variable, and ECG amplitude values as independent (predictor) variables. (**b**) shows the result of the stepwise regression (corrected for body height) with agreeableness as dependent variable, and ECG amplitude values, as well as body height, body weight, and body mass index as independent variables. The analogous regression for positive emotion is shown in (**c**).

Whereas the last section investigated relations between a specific ECG amplitude measure (the 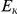 values) and personality parameters (as measured with the NEO inventory), the following section investigates whether ECG amplitude values can be used to predict NEO personality scores. To validate our computational method, we first computed stepwise regressions with body height, body weight, as well as body mass index (BMI) as dependent variables, and ECG amplitude values as independent variables (Bonferroni-corrected significance level was 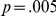, as for all regressions reported in the following section, see *Methods*). These computations indicated significant correlations for height (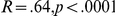, Figure 3a), weight (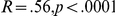), and BMI (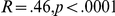; see Table S1 for regression models). Cross-validated regression coefficients (see *Methods* for cross-validation) were 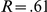 for body height, 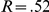 for body weight, and 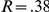 for BMI (all 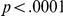). Thus, as expected, ECG amplitude values well predicted body height, weight, and BMI.

Subsequently, analogous stepwise regressions were performed with the standardized scores of the NEO scales as dependent variables, and ECG amplitudes (as well as height, weight, and BMI) as independent variables. The regression for agreeableness showed both a significant regression model (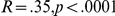), as well as a cross-validated regression coefficient of 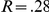 (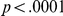, see *Methods* for cross-validation). This regression model indicated body height as one out of six predictor variables (in addition to five ECG waves, see Table S2a), due to a significant (and unexpected) correlation between agreeableness and body height in our sample (linear regression: 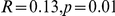). To remove this contribution of body height to the explanation of agreeableness with ECG waves, the residuals of the linear regression of agreeableness and body height were used as dependent variable in a stepwise regression, with the ECG amplitude values (as well as height, weight, and BMI) as independent variables. This analysis indicated a significant correlation between ECG amplitudes and agreeableness (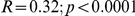, cross-validated correlation coefficient 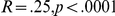), with the contribution of body height removed (Figure 3b, see Table S2b for regression model). Stepwise regressions with neuroticism or extraversion as dependent variables did not yield significiant cross-validated correlations.

Likewise, stepwise regressions were also performed with the standardized scores of the NEO facets (positive emotion, warmth, and tendermindedness) as dependent variables, and ECG amplitudes (as well as height, weight, and BMI) as independent variables. Here, the regression for positive emotion showed a significant regression model (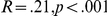, Figure 3c, see Table S2c for regression model), as well as a cross-validated regression coefficient of 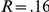 (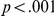).

The analogous stepwise regressions performed with the standardized scores of the NEO scales and facets as dependent variables, and the HRV parameters as independent variables (instead of ECG amplitudes) indicated a marginally significant positive correlation for positive emotion and high frequency power of the HRV (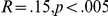, cross-validated regression coefficient: 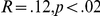). The analogous regressions with tendermindedness or warmth as dependent variables did not yield significiant cross-validated correlations.

To substantiate the relations between ECG amplitudes and agreeableness as well as positive emotion, we also performed discriminant function analyses (DFAs): Participants were split into quartiles based on the standardized NEO scores, separately for agreeableness or positive emotion (balanced for gender). Then, separately for agreeableness and positive emotion, DFAs were computed with the two groups (upper and lower quartile of agreeableness, or of positive emotion scores, respectively) as dependent variable, and the absolute ECG amplitude values, as well as body height, weight, and BMI, as predictor variables (see also Methods). The DFAs indicated significant classifications, for both agreeableness (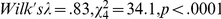, 66% cross-validated cases) and positive emotion (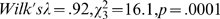, 60% cross-validated cases; see Table S3 for standardized canonical discrimination coefficients).

## Discussion

Our results reveal that ECG amplitude patterns correlate with the personality of an individual (as measured with a NEO questionnaire): *Firstly*, individuals with lower 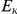 values showed higher neuroticism, and lower positive emotion, as well as a tendency towards lower extraversion (compared to individuals with higher 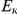 values). Note that the six items that showed the largest differences between groups (in terms of effect size) were characterized by words such as ‘light-hearted’, ‘cheerful’ and ‘happiness’. This supports the notion that that individuals with low 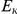 values have fewer experiences of positive feelings (such as cheerfulness and happiness), corroborating that low 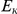 values correlate with flattened affectivity [6]. *Secondly*, cross-validated stepwise regressions, and cross-validated discriminant function analyses (DFAs) indicated that ECG amplitude values can be used to predict agreeableness and positive emotion.

With regard to the heart rate variability (HRV) measures, individuals with lower 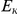 values showed lower high frequency (HF) power, higher low frequency (LF) power, a higher LF/HF ratio, and nominally (though statistically not significant) lower RMSSD. This pattern of HRV parameters replicates our previous observations [6] (see also Introduction), and resembles the HRV pattern previously reported in studies investigating patients with major depression [15] or pathologic anxiety [41], [42], that is, in individuals with disorders that are characterized by higher neuroticism, and frequent phases of absence of positive emotions. For example, a recent meta analysis [15] reported that depressed patients exhibited reduced HRV, reduced HF spectral power of the HRV, and an increased LF/HF ratio.

LF and HF components of the HRV provide information about the autonomic tone of an individual (i.e. about the strength of sympathetic and parasympathetic activation): The LF component is taken to reflect sympathetic or perhaps both sympathetic and parasympathetic effects, and the HF component is taken to reflect mainly parasympathetic effects [32], [15]. The LF/HF ratio is taken to reflect autonomic balance (i.e., the balance or predominance of the sympathetic/parasympathetic branches of the autonomic nervous system) [32]. Thus, the present results support previous observations that autonomic tone and autonomic balance differ between individuals with high and low 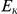 values [6]. Notably, the autonomic balance of an individual has effects on regional heart activity: Autonomic imbalance is associated with lateralization of central autonomic drive [21], and such lateralized autonomic drive leads to a modulation of signal transduction mechanisms on the anterior and posterior heart surface, as well as to a modulation of atrioventricular (AV) and sinoatrial (SA) node conductivity: As mentioned in the Introduction, the right cardiac nerve innervates the anterior surface of the heart (including the SA node, the AV node, and the anterior surfaces of the right and left ventricles), whereas the left cardiac nerve innervates the posterolateral surface of the heart (including the AV node and the posterior surfaces of the right and left ventricles [42], [24], [25]. Thus, it is conceivable that the autonomic imbalance of individuals with low 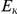 values results in a left-right imbalance in autonomic drive across the surface of the heart, which modulates the electrophysiological homogeneity of ventricular de- and repolarization [43].

Previous research also suggested that negative feelings, such as discontent, anger and competitiveness, are associated with autonomic functions involved in signal transduction mechanisms that might lead to left ventricular hypertrophy [44], and it is possible that such a morphological factor accounts partly for the ECG pattern described in the present study. However, we [6] have previously reported that serum concentrations of N-terminal-pro brain natriuretic peptide (NT-pro BNP) [45] do not correlate with 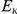 values. Because NT-pro BNP is associated with left ventricular hypertrophy, and correlates with left ventricular mass [46], this suggests that gross morphological differences between the hearts of individuals with higher and lower 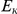 values are unlikely in our non-clinical population, at least with respect to left ventricular mass. This assumption is corroborated by the observation that, in the present study, neither QRS axis orientation, nor body height, body weight, or BMI differed between individuals with higher and lower 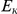 values. This strongly suggests that the 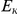 values are not simply determined by the anatomy of an individual, but rather reflect aspects of regional cardiac activity. In this regard, it is also important to note that the 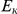 values do not simply reflect that ECG amplitudes are generally “larger” or “smaller”, but that the 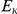 values are the result of a computation of ECG amplitude ratios (see Figure 1): For example, a larger amplitude of the R-wave in lead III results in a higher 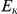 value, whereas a larger amplitude of the RS-wave in aVL results in a lower 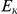 value.

Similarly, with regard to the prediction of agreeableness and positive emotion (computed based on all measured ECG amplitude values), the final regression models did not include body weight, body height, nor body-mass index as predictor variables (see Tables S2b, c). That is, the prediction of agreeableness and positive emotion based on ECG amplitude values was not simply determined by body weight, body height, or body-mass index.

As mentioned in the Introduction, regional cardiac activity is modulated by the endocrine and neurological communication between the central nervous system and the cardiac nerve plexus. Neural mechanisms underlying modulations of regional cardiac activity include personality-characteristic autonomic innervation (including forebrain asymmetries in autonomic innervation) [21], as well as personality-characteristic endocrine activity [12]. Note that the neurological axis modulating heart activity reaches up to the forebrain, including the amygdala, the insular cortex and obitofrontal cortex [20]. These structures have been implicated in emotion, empathy, and personality, and both functional and structural differences in these structures have been shown as a function of an individual's personality [2], [4], [1], [3], [6], [47], [48]. Such personality-characteristic modulations of the neurological axis between the brain and the heart are consistent with HRV studies showing relations between HRV and depression [31], [14], [15]. Our data suggest that such personality-characteristic dispositions modulate the input into the cardiac nerve plexus, with effects on regional cardiac activity and thus on ECG amplitudes. For example, it has been shown that lateralized autonomic drive leads to alterations of signal transduction mechanisms on the anterior and posterior heart surface [43] that result in electrophysiological inhomogeneities of ventricular (re)polarization [43]. Such characteristics of regional cardiac activity are likely to be reflected in amplitude patterns of high-resolution ECGs. The exact mechanisms that lead to personality-characteristic modulations of regional heart activity, however, remain to be specified.

The present results are important for two reasons: *Firstly*, due to the above-mentioned relations between personality and cardiovascular diseases [49], [50], [51], [52], further research is likely to identify cardiac signatures characteristic for individuals at risk for such diseases. For example, using the methodological and statistical approach of the present study, future studies could compare ECG amplitude values between healthy individuals with Type A or Type D personality, and normal controls, to identify pre-clinical ECG amplitude signatures of individuals at risk for coronary heart disease or chronic heart failure. It is even conceivable that cardiac signatures can be identified that are characteristic for other chronic somatic diseases involving an affective personality component. For example, we are currently investigating ECG amplitude signatures of patients with autoimmune diseases such as Morbus Crohn or type 1 diabetes, with the aim to identify ECG signatures that can potentially be used for pre-clinical diagnosis of such diseases. A similar approach could be taken with regard to depression, particulary due to the finding that individuals with lower 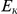 values showed increased neurotisicm, and reduced positive emotion.

*Secondly*, research on relations between ECG amplitude patterns and personality might provide cardiac indices that are potentially suited to assess personality. So far, empirical research on personality has relied predominantly on personality questionnaires. Such paper-pencil measures of personality have the advantage that they are capable of obtaining attitudes and behaviours of an individual. However, paper-pencil tests face the problem of several sources of potential subjective bias such as socially desirable responding, inaccuracies in self-perception, self-favouring tendencies, self-deception, and moralistic bias [33], [34], [35], [36], [37]. Notably, long-term emotional dispositions also have long-term peripheral-physiological effects [17], [2], [14], [8], [16], [11], [15], [12], [13]. Because such effects are more directly related to an individual's personality, identifying such effects can provide physiological indices of personality that are objective in the sense that they can hardly be influenced voluntarily by an individual (although they may be influenced by factors unrelated to personality).

### Conclusions

Our results show that the cardiac amplitude signature captured by the 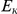 values is related to neuroticism and positive emotion, thus corroborating the notion that low 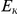 values reflect flattened affectivity. In addition, our results also reveal that agreeableness (as measured with the NEO-FFI) can be predicted to a moderate degree by ECG amplitude patterns. The findings give rise to future research investigating relations between ECG amplitude patterns and other personality measures (such as other NEO-PI-R scales and facets, or scales of other personality inventories) [53]. Our results also open perspectives for a more efficient assessment of personality using cardiac measures, with the advantage that such biological measures are less prone to several sources of subjective bias (although they can potentially be biased by other factors, this remains to be specified). The present results also give rise to identify ECG signatures characteristic for affective disorders and personality disorders characterized by increased neuroticism, and reduced positive emotion (such as depression, or schizoid personality disorder). Similarly, due to the relations between personality on the one side, and endocrine, autonomic, as well as immune system activity on the other [14], [16], [15], [12], [11], [10], [13], [8], [9], the present results also open perspectives for investigations on cardiac amplitude signatures characteristic for individuals at risk for cardiovascular disease, as well as for cardiac signatures characteristic for chronic somatic diseases involving a personality-specific affective component [14], [17]. Identification of such ECG signatures could lead to more efficient risk-stratification, risk-prevention models, and pre-clinical diagnostics.

## Supporting Information

Table S1
**Models resulting from stepwise linear regressions with absolute values of all ECG amplitude waves (see Methods) as independent (predictor) variables, and body height (a), body weight (b), and body mass index (c) as dependent variables.** 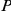: P-wave amplitude, 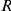: R-wave amplitude, 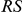: RS-wave amplitude, 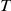: T-wave amplitude. Subscript indicates the ECG lead.(PDF)

Table S2 **Regression models. (a) Model resulting from stepwise linear regressions with absolute values of all ECG amplitude waves (see Methods), as well as body height, weight, and BMI, as independent (predictor) variables, and agreeableness (standard scores) as dependent variable.** Note that this regression model included body height as a predictor variable (due to a significant correlation between body height and agreeableness in our sample, see main text). To remove this contribution of body height to the explanation of agreeableness with ECG waves, the residuals of a linear regression with agreeableness as dependent variable, and body height as independent variable were used for a stepwise regression with the ECG amplitude values (as well as height, weight, and BMI) as independent variables. (b) shows the model of resulting from this regression (in which the explanation of agreeableness by means of ECG amplitude values is corrected for body height). (c) Model resulting from stepwise linear regressions with absolute values of all ECG amplitude waves (see Methods), as well as body height, weight, and BMI, as independent (predictor) variables, and positive emotion (T-scores) as dependent variable. Abbrevations of ECG waves as in Table S1.
(PDF)

Table S3 **Standardized canonical discrimination coefficients.** The dependent variables of the discriminant analyses were upper and lower quartiles of standard agreeableness scores (a), or upper and lower quartiles of standard positive emotion scores (b), predictor variables were the absolute ECG amplitude values, as well as body height, weight, and BMI. Abbrevations of ECG waves as in Table S1.
(PDF)

Table S4
**Items for which the scores between the two** 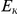 **groups (** 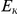 **values above and below the median of** 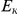 **values) differed with effect sizes of Cohen's** 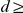 **.2. A plus sign indicates that scores were higher (“agree”) for individuals with lower** 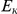 **values, the minus sign indicates that scores were lower (“disagree”) for individuals with lower** 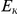 **values.** For example, individuals with lower 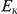 values had higher scores agreeing to the statement of not being a cheerful optimist. Cohen's 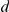 was computed using Hedges' bias correction. When comparing the scores of these items between groups using two-samples 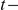
tests, 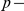
values were 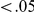 in each of the tests.(PDF)
