# Supplementary material for: Cardiac Signatures of Personality
Source: PLoS One. 2012 Feb 21;7(2):e31441. doi: 10.1371/journal.pone.0031441 (PMC3283631; doi:10.1371/journal.pone.0031441)
Supplement: Table S1 — Models resulting from stepwise linear regressions with absolute values of all ECG amplitude waves (see Methods) as independent (predictor) variables, and body height (a), body weight (b), and body mass index (c) as dependent variables. : P-wave amplitude, : R-wave amplitude, : RS-wave amplitude, : T-wave amplitude. Subscript indicates the ECG lead. (PDF) [file pone.0031441.s001.pdf]

(a) Height

$(Constant)$	159.83280
Wave$_{lead}$	coefficient
T_{Vmax}	0.01509
RS_{V5}	0.00348
T_{RIII}	0.02264
T_{RII}	-0.01249
P_{V3}	-0.03987
P_{V1}	0.02410
R_{RIII}	-0.00632
RS_{avF}	0.00523

(b) Weight

$(Constant)$	53.98237
Wave$_{lead}$	coefficient
RS_{V5}	0.00620
R_{avL}	0.00852
RS_{V2}	-0.00278
T_{avL}	0.09060
T_{avL}	-0.06621
T_{V2}	0.01421

(c) BMI

$(Constant)$	20.15687
Wave$_{lead}$	coefficient
RS_{RI}	0.00241
T_{V6}	0.00417
T_{avF}	-0.00443
P_{RIII}	-0.00373
R_{avL}	0.00199

Models resulting from stepwise linear regressions with absolute values of all ECG amplitude waves (see Methods) as independent (predictor) variables, and body height (a), body weight (b), and body mass index (c) as dependent variables. P: P-wave amplitude, R: R-wave amplitude, RS: RS-wave amplitude, T: T-wave amplitude. Subscript indicates the ECG lead.
