# Supplementary material for: Cardiac Signatures of Personality
Source: PLoS One. 2012 Feb 21;7(2):e31441. doi: 10.1371/journal.pone.0031441 (PMC3283631; doi:10.1371/journal.pone.0031441)
Supplement: Table S2 — Regression models. (a) Model resulting from stepwise linear regressions with absolute values of all ECG amplitude waves (see Methods), as well as body height, weight, and BMI, as independent (predictor) variables, and agreeableness (standard scores) as dependent variable. Note that this regression model included body height as a predictor variable (due to a significant correlation between body height and agreeableness in our sample, see main text). To remove this contribution of body height to the explanation of agreeableness with ECG waves, the residuals of a linear regression with agreeableness as dependent variable, and body height as independent variable were used for a stepwise regression with the ECG amplitude values (as well as height, weight, and BMI) as independent variables. (b) shows the model of resulting from this regression (in which the explanation of agreeableness by means of ECG amplitude values is corrected for body height). (c) Model resulting from stepwise linear regressions with absolute values of all ECG amplitude waves (see Methods), as well as body height, weight, and BMI, as independent (predictor) variables, and positive emotion (T-scores) as dependent variable. Abbrevations of ECG waves as in Table S1. (PDF) [file pone.0031441.s002.pdf]

**(a)** Agreeableness	
$(Constant)$	129.28984
Wave$_{lead}$	coefficient
Height (cm)	-0.19594
RS_{avR}	0.01943
RS_{V6}	-0.00889
P_{V4}	0.27464
P_{V5}	-0.07347
P_{V3}	-0.12404

**(b)** Agreeableness (corrected)	
$(Constant)$	-10.30131
Wave$_{lead}$	coefficient
RS_{avR}	0.01952
RS_{V6}	-0.00898
P_{V4}	0.27189
P_{V5}	-0.07010
P_{V3}	-0.12432

**(c)** Positive emotion	
$(Constant)$	45.31564
Wave$_{lead}$	coefficient
T_{RI}	0.01861
T_{V2}	-0.00781
RS_{V2}	0.00170

Regression models. (a) Model resulting from stepwise linear regressions with absolute values of all ECG amplitude waves (see Methods), as well as body height, weight, and BMI, as independent (predictor) variables, and agreeableness (standard scores) as dependent variable. Note that this regression model included body height as a predictor variable (due to a significant correlation between body height and agreeableness in our sample, see main text). To remove this contribution of body height to the explanation of agreeableness with ECG waves, the residuals of a linear regression with agreeableness as dependent variable, and body height as independent variable were used for a stepwise regression with the ECG amplitude values (as well as height, weight, and BMI) as independent variables. (b) shows the model of resulting from this regression (in which the explanation of agreeableness by means of ECG amplitude values is corrected for body height). (c) Model resulting from stepwise linear regressions with absolute values of all ECG amplitude waves (see Methods), as well as body height, weight, and BMI, as independent (predictor) variables, and positive emotion (T-scores) as dependent variable. Abbrevations of ECG waves as in Supplementary Table 1.
