# Supplementary material for: Cardiac Signatures of Personality
Source: PLoS One. 2012 Feb 21;7(2):e31441. doi: 10.1371/journal.pone.0031441 (PMC3283631; doi:10.1371/journal.pone.0031441)
Supplement: Table S3 — Standardized canonical discrimination coefficients. The dependent variables of the discriminant analyses were upper and lower quartiles of standard agreeableness scores (a), or upper and lower quartiles of standard positive emotion scores (b), predictor variables were the absolute ECG amplitude values, as well as body height, weight, and BMI. Abbrevations of ECG waves as in Table S1. (PDF) [file pone.0031441.s003.pdf]

(a) Agreeableness

Wave$_{lead}$	coefficient
P_{avR}	0.47351
RS_{avR}	0.82592
RS_{V6}	-0.54983
T_{V6}	-0.51205

(b) Positive Emotion

Wave$_{lead}$	coefficient
T_{V2}	-0.73216
P_{V6}	0.70459
RS_{Vmax}	0.59898

Standardized canonical discrimination coefficients. The dependent variables of the discriminant analyses were upper and lower quartiles of standard agreeableness scores (a), or upper and lower quartiles of standard positive emotion scores (b), predictor variables were the absolute ECG amplitude values, as well as body height, weight, and BMI. Abbrevations of ECG waves as in Supplementary Table 1.
