# Supplementary material for: Cardiac Signatures of Personality
Source: PLoS One. 2012 Feb 21;7(2):e31441. doi: 10.1371/journal.pone.0031441 (PMC3283631; doi:10.1371/journal.pone.0031441)
Supplement: Table S4 — Items for which the scores between the two groups ( values above and below the median of values) differed with effect sizes of Cohen's .2. A plus sign indicates that scores were higher (“agree”) for individuals with lower values, the minus sign indicates that scores were lower (“disagree”) for individuals with lower values. For example, individuals with lower values had higher scores agreeing to the statement of not being a cheerful optimist. Cohen's was computed using Hedges' bias correction. When comparing the scores of these items between groups using two-samples tests, values were in each of the tests. (PDF) [file pone.0031441.s004.pdf]

Item	effect size
I don’t consider myself especially "light-hearted". (+)	.24
I am not a cheerful optimist (+)	.23
I often feel tense and jittery (-)	.23
Sometimes I bubble with happiness (+)	.22
I rarely feel lonely or blue (-)	.21
I am a cheerful, high-spirited person (+)	.21

Items for which the scores between the two E_κ groups (E_κ values above and below the median of E_κ values) differed with effect sizes of Cohen’s $d \geq .2$. A plus sign indicates that scores were higher ("agree") for individuals with lower E_κ values, the minus sign indicates that scores were lower ("disagree") for individuals with lower E_κ values. For example, individuals with lower E_κ values had higher scores agreeing to the statement of not being a cheerful optimist. Cohen’s d was computed using Hedges’ bias correction. When comparing the scores of these items between groups using two-samples $t-$tests, $p-$values were $< .05$ in each of the tests.
